# Synthesis and characterization of polysaccharide- and protein-based edible films and application as packaging materials for fresh fish fillets

**DOI:** 10.1038/s41598-024-51163-y

**Published:** 2024-01-04

**Authors:** Evmorfia Athanasopoulou, Francesco Bigi, Enrico Maurizzi, Eva Iris Eleftheria Karellou, Christos S. Pappas, Andrea Quartieri, Theofania Tsironi

**Affiliations:** 1 Laboratory of Food Process Engineering, Department of Food Science and Human Nutrition, Agricultural University of Athens, Iera Odos 75, 11855 Athens, Greece; 2 Packtin, Via Del Chionso, 14/I, 42122 Reggio Emilia, RE Italy; 3 Department of Life Science, University of Modena and Reggio Emilia, Via John Fitzgerald Kennedy 17/I, 42122 Reggio Emilia, RE Italy; 4 AVRAMAR Aquaculture SA, Athens, Greece; 5 Laboratory of Chemistry, Department of Food Science and Human Nutrition, Agricultural University of Athens, Iera Odos 75, 11855 Athens, Greece

**Keywords:** Polymers, Chemical engineering

## Abstract

The rising packaging industry together with global demand for sustainable production has increased the interest in developing biodegradable packaging materials. The aim of the study was to develop edible films based on pectin, gelatin, and hydroxypropyl methylcellulose and evaluate their applicability as biodegradable packaging materials for gilthead seabream fillets. Mechanical properties, water barriers, wettability of the films through contact angle measurement, optical, and UV–Vis barrier properties were evaluated for food packaging applications. The effective blend of polysaccharide and protein film-forming solutions was confirmed by the produced films with excellent optical properties, acceptable mechanical properties and adequate barriers to water vapor. The contact angle for pectin based and gelatin based films were higher than 90° indicating the hydrophobic films, while HPMC based films had contact angle lower than 90°. The produced films were tested as alternative and environmentally friendly packaging materials for gilthead seabream fillets during refrigerated storage. All tested packaging conditions resulted in similar shelf-life in packed gilthead seabream fillets (i.e. 7–8 days at 2 °C). The results showed that the developed films may reduce the use of conventional petroleum-based food packaging materials without affecting the shelf-life of fish.

## Introduction

The packaging field contributes significantly to the carbon footprint. Packaging materials represent the largest category of plastic waste, almost 50% of global plastic waste comes from the packaging field^1^. From 1950 to 2015 recycling plastic accounted for only 9% of plastic waste. According to Plastic Waste Management Market Size, global plastic waste's compound annual growth rate (CARG) is estimated as 5.4% from 2022 to 2030^2^. Edible food packaging has been evaluated as an alternative, environmentally friendly method to maintain freshness in foods and extend the shelf-life. Under this context, conventional petroleum-based packaging materials can be replaced or substituted by edible and biodegradable films^3^.

According to the literature, edible packaging materials are divided into films (commonly called membranes) and coatings. The main difference between films and coatings is that the films are initially solubilized as solid packaging sheets and then wrapped around food, while coating is applied directly on the food surface by an immersion step or spraying^3^. The edible films used for food packaging are edible biopolymers, such as lipids, polysaccharides, and proteins combined with other ingredients which are acceptable for human consumption and derived from non-common sources^4^.

Edible films and coatings can be categorized into three main groups according to the type of material from which they are derived, i.e. polysaccharides, proteins, and lipids. Polysaccharides are widely available and ones of the most widely used are cellulose derivatives and pectin.

Cellulose is a polysaccharide commonly used in food applications, which has been reported as alternative food packaging material for the replacement of conventional petrochemical based plastics^5^. HPMC can be produced by cellulose modification by an alkaline treatment. HPMC can be produced using a 18% sodium hydroxide solution in purified wood pulps. Methyl and hydroxypropyl ether groups are introduced into cellulose, due to the reaction of alkali cellulose with propylene oxide followed with reaction with methyl chloride. HPMC is an odorless and tasteless compound, and thus forms films with neutral organoleptic characteristics, considering odor and taste^6^.

Pectin is a cell wall polysaccharide, which is essential for primary cell wall extension and plant growth. Pectin may be extracted from citrus peel, exhibits high solubility in water and is insoluble in organic systems. Pectin mainly consists of galacturonic acid (70%), forming linear anionic backbone chains without side parts and non-anionic regions. The free carboxyl groups in the pectin structure provide a solution with acidic pH. Pectin has been reported as an appropriate compound for packaging film formation, mainly due to its compatibility with other natural polysaccharides, proteins or lipids^7^.

Proteins are used in packaging field as they have the ability to form biodegradable films. Films based on proteins have satisfactory mechanical and adequate gas barrier properties^8^.

Gelatin is reported as an alternative biopolymer, appropriate for food packaging applications, with the aim to enhance the functionality of packed products. It is considered as a water-soluble protein, obtained from partial hydrolysis of collagen. Because of its hydroscopic nature, gelatin-based films tend to dissolve in environments with increased moisture levels. The overall properties of gelatin-based films are dependent on molecular weight distribution and amino acid synthesis, directly related to the gelatin source, the extraction method and processing conditions^9^. Gelatin as raw material has no antimicrobial properties, however, antioxidant and antimicrobial compounds such as citrus essential oils and carvacrol may be incorporated into gelatin-based film forming solutions^8^.

Polysaccharides and protein blend materials have been tested for their biocompatibility to form packaging films. Generally, in composite films, each biopolymer gives its own unique characteristics, so the properties of the films are improved compared to single-material systems, considering the compatibility between the used biopolymers. There are three possible types of biopolymers that can improve the properties of single edible films: protein–protein, polysaccharide–polysaccharide and protein-polysaccharide^10^. Gelatin has low thermal and weak mechanical properties, while HPMC has gelation properties. In order to overcome these disadvantages HPMC and gelatin can be blended to form a continuous matrix^11^. Gelatin and pectin could produce value–added packaging materials since having excellent film forming properties and produce compatible films, as reported in the literature^12,13^.

Fish and seafood products are important commodities due to their high nutritional and commercial value. The high water activity values (0.95–0.98) of fish and seafood together with the increased concentrations of nutrients result in the perishability of these products and their limited shelf-life. Globally, 35% of the total fish production is wasted before consumption^14^. For this reason, it is crucial to develop packaging materials that are environmentally friendly and can adequately maintain the freshness of fish products^15^.

The fabrication of HPMC, pectin, and gelatin by solvent casting method as separate films has been studied but their compatibility and characterization as composite films has not been yet justified in the literature^9,10,12,16^. Gelatin, pectin, and HPMC are three biopolymers which are dissolved in water and their films are characterized as hydrophilic and with high water content^17,18^. Although high water content is linked with biodegradability of the films, this may result in limitations in their application as food packaging materials, especially for foods with high moisture content. By mixing different proteins and polysaccharides, the chains of the polymers matrix are reorganized to the air-side interface, in order to form a more hydrophobic surface^19,20^. Another potential difficulty is the testing of the materials in sensitive food products, their applicability as alternative packaging systems has not been yet reported for fish products^10^.

The aim of the study was to develop and characterize composite films based on pectin, gelatin, and HPMC. The films were evaluated as packaging materials based on their mechanical properties, optical properties-and UV–Vis transmittance, water barriers, and hydrophilicity. Three different film formulations (HPMC-gelatin, HPMC-pectin, pectin-gelatin) were evaluated for their applicability as alternative packaging materials for chilled gilthead seabream (*Sparus aurata*) fillets during refrigerated storage at 2 °C as compared to a conventional polyvinyl chloride (PVC) film.

## Results and discussion

### FT-IR analysis

**Figure 1:**
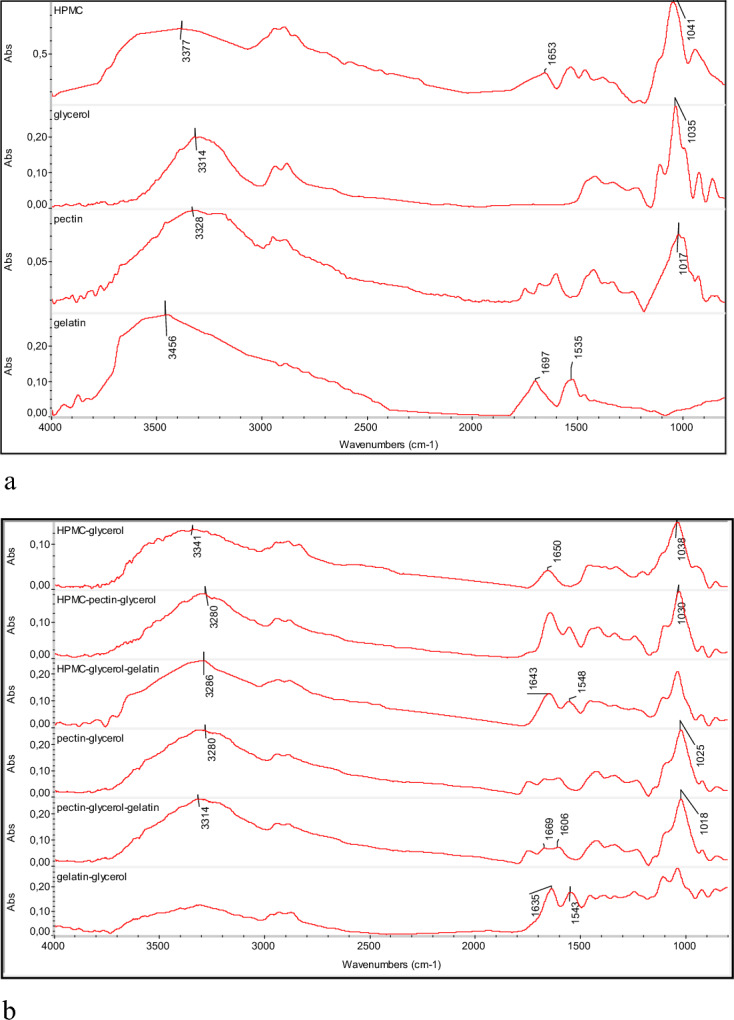
(**a**) The FT-IR spectra (4000–800 cm^−1^) of HPMC, glycerol, pectin and gelatin. (**b**) The FT-IR spectra (4000–800 cm^−1^) of HPMC-glycerol, HPMC-pectin-glycerol, HPMC-glycerol-gelatin, pectin-glycerol, pectin-glycerol-gelatin, gelatin-glycerol.

The FT-IR spectra are presented in Fig. 1a,b. A main difference exists between HPMC, glycerol and HPMC-glycerol spectra and was found at 3314–3377 cm^−1^ and it has been associated with the O–H stretching^21,22^. This difference may be related to the intermolecular hydrogen bonds between the -OH of HPMC and glycerol.

In the same spectral region and for the same reason, differentiation occurred (3341–3280 cm^−1^) between the pectin and the films were made of HPCM-glycerol, and the HPMC-pectin-glycerol FT-IR spectra. Moreover, for the same substances, differentiation also appeared in the peak 1017–1038 cm^−1^ (C–O stretching)^21–23^. According to the literature, pure HPMC films showed a strong absorption band at 1049 cm^−1^ and 945 cm^−1^ linked with ν_as_(C–O–C) of pyranose ring skeletal vibrations and ether linkages, respectively. Peaks at both wavelengths indicate the presence of ether groups in the HPMC structure^19^.

Shivangi et al. developed pectin films and the peaks of pure pectin films were at − 3330 cm^−1^ (O–H stretching vibration), 2934 cm^−1^ (C–H vibrations), 1740 cm^−1^ (C=O vibrations), 1615 cm^−1^ (–COO vibrations), 1440 cm^−1^ (CH_3_ plane bend)^24^.

In spectra of HPMC and HPMC-glycerol a peak appeared at 1650 cm^−1^ which is due to the bending vibration of absorbed water^25^. In gelatin spectrum, the peaks were at 1697 (amide I) and 1535 cm^−1^ (amide II)^26^. These peaks shifted to 1643 and 1548 cm^−1^ in the films based on HPMC-glycerol-gelatin, probably due to the hydrogen bonds that developed. Wang et al. investigated the synthesis of HPMC films grafted by gelatin and they used glycerol as a plasticizer. The FT-IR analysis showed that the hydroxyl groups that are present in the HPMC and gelatin molecules had peak at 3280 cm^−1^. Pure HPMC films had peaks at 3460 and 1040 cm^−1^ due to the stretching vibration of the hydroxyl groups and ether bond groups, respectively. When the HPMC films grafted with gelatin the peaks moved at 1547 cm^−1^ and 1240 cm^−1^. These two peaks are the characteristic peaks of amide II band (relating to the bending vibration of N–H) and amide III band (relating to stretching vibration of C–N), and they are attributed to peptide bond of gelatin. The authors also pointed that by adding gelatin in HPMC matrix the intensity of peaks near 1040 cm^−1^ was lower while the intensity of peaks near 1645 cm^−1^ was higher^27^. This result is also observed in the present study as it is shown in Fig. 1a,b.

The FT-IR spectra pectin, glycerol and pectin-glycerol differentiated in spectral region 3314–3280 cm^−1^ and at 1017–1035 cm^−1^. The gelatin spectrum appeared a peak at 3456 cm^−1^ which corresponds to N–H stretching. The pectin-glycerol spectrum showed a peak at 3280 cm^−1^ (O–H stretching). We observed that the pectin-glycerol-gelatin spectrum presents an absorption at 3314 cm^−1^. This shift correlated with hydrogen bonding. Another differentiation between pectin-glycerol and pectin-glycerol-gelatin spectra presented in the spectral region 1018–1025 cm^−1^ (C–O stretching). Ezati et al. studied the chemical interactions between pectin and gelatin using FT-IR analysis. They pointed peaks at 3248, 2936 and 2880 cm^−1^ due to O–H stretching and the intermolecular or intramolecular H-bonding, and C–H stretching vibration of methylene and methyl groups that were appeared in pectin. Peak at 1454 cm^−1^ was also due to pectin’s carboxyl groups. Peaks at 1634, 1548 and 1236 cm^−1^ were due to the amide I (C=O stretching), amide II (N–H stretching) and amide III (C–N and N–H stretching) of gelatin^12^.

Finally, the gelatin, glycerol and gelatin-glycerol presented two different peaks at 1697–1635 cm^−1^ (amide I) and a smaller at 1543–1535 cm^−1^ (amide II), possibly due to hydrogen bonding.

From the above discussion it is concluded that the complexes HPMC-glycerol, HPMC-gelatin-glycerol, pectin-gelatin, pectin-glycerol-gelatin and gelatin-glycerol have been formed in the developed packaging materials.

### Thickness and mechanical properties

The films used as packaging materials should have adequate mechanical properties to protect the food from mechanical stresses during storage and handling. Elastic modulus (MPa), tensile strength (MPa), and elongation at break (%) can describe the integrity of the films under certain circumstances^28^. The mechanical properties and the thickness of the films are reported in Table 1. The thickness of the films in this study ranged between 31.72 μm and 35.31 μm. The mechanical properties were significantly dependent on the thickness of the films, as indicated in Table 1 (p < 0.05).

**Table 1:** Thickness, tensile strength (TS), elastic modulus (EM), and elongation at break (EB) of one-compound and composite films based on pectin, HPMC, and gelatin.

Type of film	Thickness (μm)	Elastic modulus (MPa)	Tensile strength (MPa)	Elongation at break (%)
Pectin	34.82 ± 8.21^a^	66.71 ± 6.71^a^	5.59 ± 1.50^a^	13.55 ± 2.05^c^
HPMC	33.33 ± 0.81^a^	107.00 ± 15.79^c^	7.63 ± 1.57^b^	29.22 ± 4.49^e^
Gelatin	31.72 ± 5.95^a^	84.87 ± 14.28^b,c^	3.93 ± 0.91^a^	17.91 ± 2.81^d^
Pectin-HPMC	35.07 ± 2.68^a^	267.51 ± 41.53^d^	4.65 ± 0.83^a^	5.63 ± 0.96^a^
Pectin-gelatin	32.49 ± 5.95^a^	42.93 ± 11.29^a^	3.19 ± 0.73^a^	16.15 ± 2.08^d^
HPMC-gelatin	35.31 ± 4.89^a^	106.12 ± 39.82^b,c^	8.56 ± 1.77^b^	7.29 ± 1.44^b^

^a–e^Different superscripts in the same column indicate statistically significant differences.

Elastic modulus represents the stiffness of a film^28^. The results showed that films with pectin had lower elastic modulus compared to other films (66.71 ± 6.71 MPa for pectin films and 42.93 ± 11.29 MPa for pectin-gelatin film) which means pectin films were more flexible^29^. While films based on HPMC had the highest values (107.00 ± 15.79 MPa for HPMC-based films and 267.51 ± 41.53 MPa and 106.12 ± 39.82 MPa, for pectin-HPMC and HPMC-gelatin films, respectively). Asfaw et al. tested the mechanical properties of pectin films (3–5% w/v). They used glycerol as plasticizer in the range of 15–25%. They found pectin films had elastic modulus from 59.53 to 67.67 MPa, depending on the concentration of pectin and glycerol. This can be explained by the intermolecular hydrogen bonds between glycerol and pectin^28^. As far as HPMC, similar results were reported by Mahadevaiah et al. for HPMC films with different concentrations of glycerol and polyethylene glycol as plasticizers, with elastic modulus ranging from 1654.75 to 20.46 MPa^18^.

According to Table 1, the films which were based on one biopolymer had higher tensile strength and elongation at break compared to composite films. The chemical reactions between the polymer chains of different polymers influence the mechanical properties of the produced films^28^. Pure pectin films had higher tensile strength in comparison with composite films based on pectin-HPMC and pectin-gelatin but without statically differences (5.59 ± 1.50, 4.65 ± 0.83 and 3.19 ± 0.73 MPa, respectively). Films based on HPMC had higher tensile strength compared to all films. Tedesco et al. produced films based on HPMC, CMC and starch and reported mechanical properties, where HPMC films showed higher values^16^. According to the literature, composite films with fillers with micron size compounds have lower values on mechanical properties compared to films which are produced with smaller size fillers as nano-size compounds. This may be attributed to nano-size fillers which fill the empty areas in the matrix and build stronger interfacial bonding with the matrix^30^. In the present study, all used materials had similar size and thus no significant differences were observed between the single compound and composite films. Films based on gelatin and using glycerol as a plasticizer generally have lower tensile strength but higher values of elongation at break (3.93 ± 0.91 MPa and 17.91 ± 0.2.81%, respectively). By adding pectin in gelatin films, the tensile strength increased but without statistical difference.

The value of elongation at break represents the film's flexibility, which depends on the polymer network^27^. The composite films had lower elongation at break compared to respective one-material films (Table 1). According to Mirzaei-Mohkan et al., the addition of different compounds in the matrix of the film can change the uniformity of the structure and lead to a decrease in the stress volume tolerance in the film^31^.

### Water vapor transmission rate and water vapor permeability

The calculation of the water barrier of food packaging materials is important for maintaining the quality and shelf-life of the products. Each food product requires a specific value of water content but in several cases, a reduced water transfer between the intern of the packaging and the environment is acceptable for the preservation of microbial contamination due to moisture exchange^19^. The water vapor transmission rate (WVTR) and water vapor permeability (WVP) of edible films is reported in Table 2. Packaging materials based on HPMC show high permeability of water and this is associated with its long hydrophilic chains. Hydroxyl groups can create hydrogen bonding with water molecules^23^. However, in this study, there are no significant differences in the WVTR between the films based on pectin, HPMC, and gelatin.

**Table 2:** Water vapor transmission rate ($\frac{{\text{g}}}{{\text{day}}}$×m^2^) and water vapor permeability (g × m^−1^ × Pa^−1^ × day^−1^).

Type of film	Water vapor transmission rate ($\frac{{\text{g}}}{{\text{day}}}$×m^2^)	Water vapor permeability (g × m^−1^ × Pa^−1^ × day^−1^)
Pectin	4307.28 ± 382.22^a^	145.87 ± 12.94^b^
HPMC	3671.72 ± 1153.65^a^	145.76 ± 45.80^b^
Gelatin	3064.33 ± 437.36^a^	102.00 ± 14.56^a^
Pectin-HPMC	3778.03 ± 506.11^a^	154.84 ± 20.74^b^
Pectin-gelatin	5439.86 ± 763.48^a^	217.81 ± 30.57^c^
HPMC-gelatin	3659.66 ± 731.83^a^	208.55 ± 40.95^c^

^a–c^Different superscripts in the same column indicate statistically significant differences.

As far as WVP, materials based on one biopolymer showed lower values of water vapor permeability compared to composite films. Specifically, gelatin films showed the lowest value (102.00 ± 14.56 g × mm/kPa × day × m^2^). Aitboulahsen et al. produced films from gelatin and pectin at concentration of 4% w/v when used alone and at 2% for each biopolymer when used in combination. Glycerol was added at 30% based on the concentration of biopolymer. The water vapor permeability was determined using the same methodology as the present study, but the relative humidity of the desiccator was 75%, instead of 90%. The results showed that the WVP of gelatin films and pectin films was 6.35 (± 0.01) and 6.18 (± 0.10) × 10^7^ g × m^−1^ × Pa^−1^ × s^**−**1^, respectively. When the film-forming solutions of gelatin and pectin were mixed to produce composite films the WVP reduced and was equal to 4.55 (± 0.23) × 10^7^ g × m^−1^ × Pa^−1^ × s^−1^^32^. The same result was observed in the present study, as presented in Table 2, as WVP was higher for composite materials compared to single-polymer films. In another study, the WVP of apple high methoxyl pectin films tested in 38 °C and 90% RH was 1.5(± 0.15) × 10^–9^ g × m^−1^ × Pa^−1^ × s^−1^, which is comparable with the present study (1.45 (± 0.12) × 10^–9^ g × m^−1^ × Pa^−1^ × s^−1^)^33^. The WVP is also increased by choosing glycerol as plasticizer. The amount and type of plasticizer can also influence the water barrier as it is used to lessen the network density. Glycerol is a hydroscopic plasticizer and therefore the water content of films is increasing as the water permeation is increasing because of the high mobility of molecules^34^.

### Moisture content (MC)

The moisture content of foods and their packaging is an important parameter as it is associated with the growth rate of microorganisms, keep the freshness of food and the desired taste and texture^35^. The moisture content of films based on pure pectin or gelatin was lower, compared to the composite materials. The moisture content of pure pectin film was 13.38% which was increased to 20.04% and 18.04% for blending with HPMC and gelatin film-forming solutions, respectively. Khodaei et al., reported higher values of moisture content of low methyl pectin due to the hydrophilic nature of pectin and the ability of pectin’s molecules to form hydrogen bonds with water molecules. However, the moisture content of gelatin was similar with the results of the present study (11.67%)^40^. Liu et al. prepared films from gelatin using 50 mL of film forming solution at concentration of 4% (w/v) and measured the moisture content of control gelatin films equal to 14%^36^. Pure HPMC films had the higher moisture content equal to 22.13%, indicating the hydrophilicity of HPMC. Similar results were observed by Malik et al. (2022) who studied the effect of three different plasticizers on HPMC films. The moisture content of HPMC films plasticized with glycerol was 19.92%^37^.

### Water solubility (WS)

The results of water solubility are presented in Table 3. Water solubility is connected with biodegradability of the films and indicates the water resistance of the films. For food that require high water resistance and have high moisture content, films with high solubility in water are not suitable packaging materials^19^. In the present study, films based on one biopolymer have higher water solubility compared to composite films, but only in gelatin films difference was statistically significant. HPMC and pectin films exhibited the highest water solubility (59.89 ± 4.83% and 60.28 ± 5.63%, respectively). HPMC films showed high water solubility, which can be attributed to hydrophilic hydroxyl groups in the HPMC films structure^38^. Cabello et al. produced pectin films with different plasticizers and determined the water solubility for different types of films. They reported that the water solubility of pectin was 9.28 ± 0.56% but when glycerol was used as plasticizer at different concentrations (0.3%, 0.5%, 1%, 3% and 5%) the water solubility of the films increased (18.35 ± 0.14%, 23.33 ± 1.27%, 49.97 ± 2.72%, 67.67 ± 1.98%, and 100%, respectively) after 12 h of immersion^29^. The water solubility of pectin films may be attributed to hydroxyl and the non-esterified carboxyl functional group of the pectin structure, which are able to build hydrogen bonds with water^39^. Gelatin films were solubilized at 44.09 ± 2.03% in water. This value was increased when HPMC and pectin were added in the gelatin-based film forming solution. Similar results were reported in the literature, i.e. 42.00 ± 0.56% by Alexandre et al.^40^ and 31.1% by De Carvalho and Grosso^41^. Ahammed et al. produced films with 8% gelatin and reported total solubility in the water^42^.

**Table 3:** Moisture content (%) and water solubility of the developed films.

Type of film	Moisture content (%)	Water solubility (%)
Pectin	13.38 ± 0.62^b^	59.89 ± 2.83^c^
HPMC	22.13 ± 1.19^e^	60.28 ± 5.63^c^
Gelatin	11.67 ± 0.73^a^	44.09 ± 2.03^a^
Pectin-HPMC	20.04 ± 1.16^d^	59.07 ± 5.14^c^
Pectin-gelatin	18.04 ± 1.05^c^	56.32 ± 3.25^b^
HPMC-gelatin	21.86 ± 1.12^e^	57.28 ± 7.59^b,c^

### Contact angle

**Figure 2:**
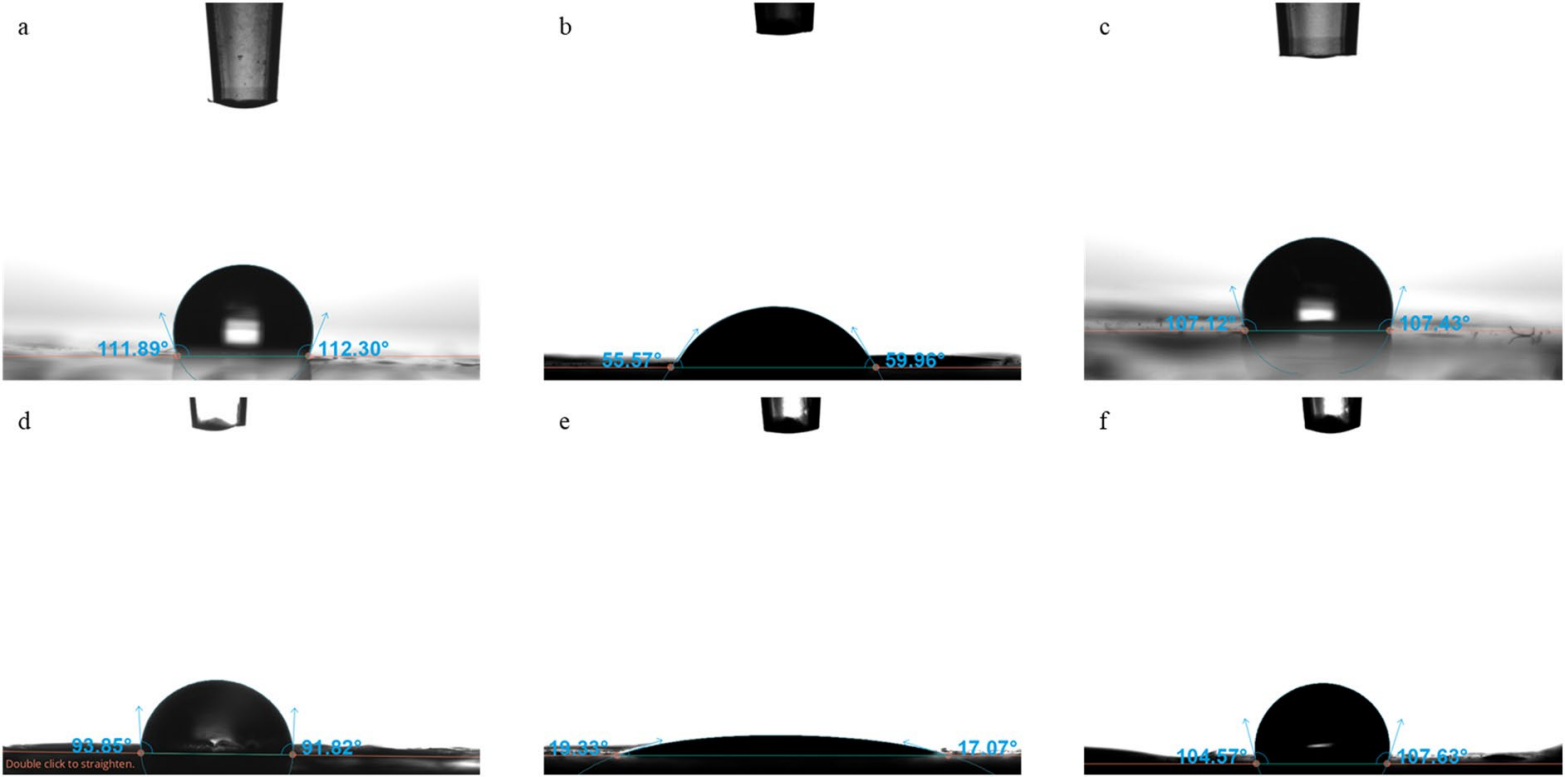
Contact angle of (**a**) gelatin based film, (**b**) HPMC based film, (**c**) pectin based film, (**d**) HPMC-gelatin based film, (**e**) pectin-HPMC based film, and (**f**) pectin-gelatin film.

For packaging materials, their ability to resist water absorption is a significant property. This property is indicated by water contact angle onto at the surface of the materials^43^. Additionally, the measurement of water contact angle provides information regarding the physicochemical interaction at the hydrogel interface^44^. Surfaces which have water contact angle lower than 90° (θ < 90°) are characterized as hydrophilic and they are partial or complete wetting, while surfaces which have water contact angle higher than 90° (θ > 90°) are characterized as hydrophobic^45^. Overall, in the present study, the film which was synthesized using HPMC, was hydrophilic (θ = 56.49 ± 1.54°), as it is shown in Fig. 2, which is in agreement with the results reported by Tedesco et al. for HPMC based films at concentration of 7.5% with sorbitol as a plasticizer (42.42 ± 3.65°)^16^. Zhang et al. produced HPMC films at 20% concentration in distilled water, carrageenan and polyethylene glycol as solvents and reported contact angle 32.07 ± 08°, which indicates that these films were more hydrophilic^46^. HPMC contains polar functional groups of oxygen and for this reason the molecular interactions are more evident, resulting in low contact angle is low^47^. Pectin and gelatin films were hydrophobic as the contact angle in that case were 107.27 ± 2.71° and 112.15 ± 2.98°, respectively, according to Table 4. The contact angle of composite films which had HPMC as one of the materials, was lower. Pectin-HPMC films showed the lowest value equal to 18.10 ± 1.03°, indicating the presence of hydrophilic hydroxyl groups. Composite films made of gelatin as one of the materials had lower contact angle compared to pure gelatin films, but they still characterized as hydrophobic. The hydrophobic character of gelatin films can be associated with the orientation of functional groups of amino acids, during the procedure of gelification. Serine, threonine, asparagine, glutamine, aspartic acid and glutamic acid are the hydrophilic amino acids on the structure of gelatin and their functional groups are placed in aqueous positions in the film-forming solution. Leucine, valine, phenylalanine, isoleucine and methionine are the hydrophobic amino acids on the structure of gelatin, and they are reoriented to the air-side interface, in order to form a hydrophobic surface^20^. A previous study was conducted by Ahammed et al. reporting the hydrophobic surface of gelatin films (θ = 10.48 ± 1.8°)^42^.

**Table 4:** Contact angle (°) of films.

Type of film	Contact angle (°)
Pectin	107.27 ± 2.17^d^
HPMC	56.49 ± 1.54^b^
Gelatin	112.15 ± 2.98^d,e^
Pectin-HPMC	18.10 ± 1.03^a^
Pectin-gelatin	106.40 ± 5.65^d^
HPMC-gelatin	93.49 ± 3.52^c^

### Color parameters

**Figure 3:**
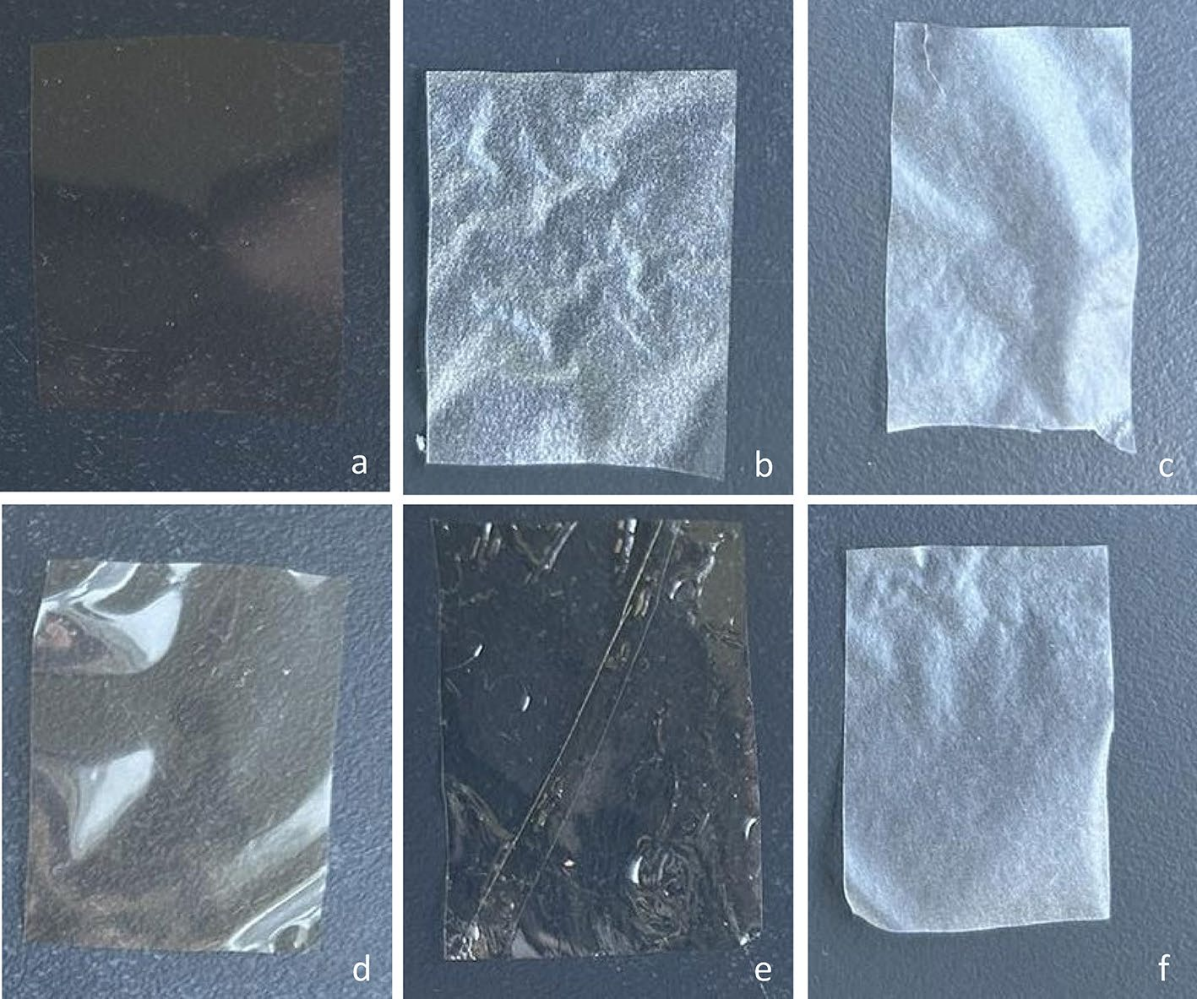
Visual appearance of (**a**) pectin, (**b**) HPMC, (**c**) gelatin, (**d**) pectin-HPMC, (**e**) pectin-gelatin, and (**f**) HPMC-gelatin based films.

The appearance and the characteristic colors of the films which are used as food packaging materials are important parameters for consumer acceptability. The appearance of films is shown in Fig. 3.

In the present study, the color of the films was investigated using instrumental (CIE L*, a* and b*) color evaluation. In Table 5, color parameters (L*, a*, b*) and total color variation (ΔΕ*) are shown. No significant differences were observed in the lightness (L*) for all types of films and the value ranged from 98.38 to 99.25.

**Table 5:** Color parameters (L*, a*, and b*) and total color variation (ΔΕ*) of films based on pectin, HPMC, gelatin and pectin-HPMC, pectin-gelatin, and HPMC-gelatin.

Type of film	L*	a*	b*	ΔΕ*
Pectin	98.38 ± 0.27^a^	− 0.21 ± 0.01^b^	2.15 ± 0.61^d^	2.42 ± 0.65^c^
HPMC	99.18 ± 0.28^b^	− 0.17 ± 0.03^a^	− 0.18 ± 0.10^a^	0.29 ± 0.22^a^
Gelatin	98.95 ± 0.20^a,b^	− 0.33 ± 0.05^c^	0.86 ± 0.31^c^	1.04 ± 0.34^b^
Pectin-HPMC	98.84 ± 0.21^a^	− 0.24 ± 0.06^b^	0.87 ± 0.30^c^	1.11 ± 0.35^b^
Pectin-gelatin	98.62 ± 0.36^a^	− 0.33 ± 0.11^c^	2.12 ± 1.02^d^	2.31 ± 1.09^c^
HPMC-gelatin	99.25 ± 0.24^b,c^	− 0.32 ± 0.11^c^	0.45 ± 0.30^b^	0.65 ± 0.45^a^

^a–d^Different superscripts in the same column indicate statistically significant differences.

The a* parameter shows the green–red shade of the film. The a* value was negative for all films which indicates the green shade of the films. Blueness-yellowness is expressed by b* parameter. The yellow shade is indicated by a positive b* value. In the present study, all films, apart from HPMC film, had positive b* value. For all produced films, the ΔΕ* values were lower than 3 and the color change was not visible to the naked eye. In general, a ΔΕ* value lower than 3 indicated the transparency of the films^48^.

Compared with pure HPMC film, a significant decrease in negative values of a* and positive values of b* and ΔΕ* was observed in HPMC-gelatin and pectin gelatin films, indicating the stronger green and yellow color of the composite films. Films with one or two biopolymers based on pectin had higher values of b* parameters indicating the yellow shade of pectin as raw material. For the same reason, films based on pectin had higher ΔΕ* value, which means higher differences from the white standard.

### UV–Vis barrier and light transmittance

The light barrier is an important parameter for the materials used in food packaging, as it expresses the barrier ability against ultraviolet–visible (UV–Vis) light. The wavelength range for the UV region is between 100 and 400 nm and split in three categories: UVA (315–400 nm), UVB (280–315 nm), and UVC (100–200 nm). The wavelength range for visible light is between 380 and 700 nm. Packaging films with good light barrier could extend the shelf-life of sensitive food products, where lipid oxidation and loss of vitamins should be prevented^32^. Table 6 presents UV–Vis light transmittance at wavelengths between 200 and 800 nm for all films.

**Table 6:** UV–Vis light transmittance (200–800 nm) of films based on pectin, HPMC, gelatin and pectin-HPMC, pectin-gelatin, and HPMC-gelatin.

Type of film	Light transmittance (%) at different wavelengths (nm)							
	200	280	350	400	500	600	700	800
Pectin	0.04 ± 0.00^c^	68.31 ± 0.86^d^	83.88 ± 1.19f.	87.05 ± 2.12^e^	88.76 ± 2.75^c^	89.48 ± 2.43^c^	89.55 ± 2.89^c^	89.64 ± 3.13^c^
HPMC	0.01 ± 0.00^a^	40.57 ± 9.52^a^	40.35 ± 9.14^a^	40.34 ± 8.82^a^	41.17 ± 8.57^a^	42.90 ± 8.33^a^	42.88 ± 8.39^a^	42.97 ± 8.36^a^
Gelatin	0.01 ± 0.00^a^	43.42 ± 1.94^b^	67.12 ± 5.12^d^	68.80 ± 6.50^c^	69.60 ± 6.85^b^	71.13 ± 6.78^b^	71.06 ± 7.03^b^	70.98 ± 7.13^b^
Pectin-HPMC	0.03 ± 0.01^b,c^	45.14 ± 0.41^b^	59.91 ± 2.63^c^	62.80 ± 3.26^b^	65.62 ± 3.71^b^	67.75 ± 3.81^b^	68.42 ± 3.94^b^	68.59 ± 4.03^b^
Pectin-gelatin	0.02 ± 0.01^a,b^	58.53 ± 2.82^c^	81.51 ± 1.76^e^	84.70 ± 2.36^d^	86.40 ± 2.76^c^	87.11 ± 2.69^c^	87.44 ± 2.83^c^	87.40 ± 2.99^c^
HPMC-gelatin	0.01 ± 0.00^a^	34.99 ± 6.79^a^	54.20 ± 2.98^b^	58.79 ± 0.70^b^	63.94 ± 1.66^b^	67.42 ± 2.59^b^	69.58 ± 3.45^b^	71.10 ± 4.18^b^

^a–f^Different superscripts in the same column indicate statistically significant differences.

The value of transmittance at 200 nm was lower than 0.1% for all films, which indicates great barrier properties at UVC. The transmittance in the visible wavelength range (400–800 nm) was high for the films, which means that the films were transparent. Specifically, for pectin films and gelatin films, the transmittance was higher than 70%, which means that all the films were clear and transparent. This is in agreement with the study reported by Esteghal et al., where gelatin and HPMC-based films were highly transparent since they exhibited low absorbance^10^.

### Microbiological evaluation of packed gilthead seabream fillets

**Figure 4:**
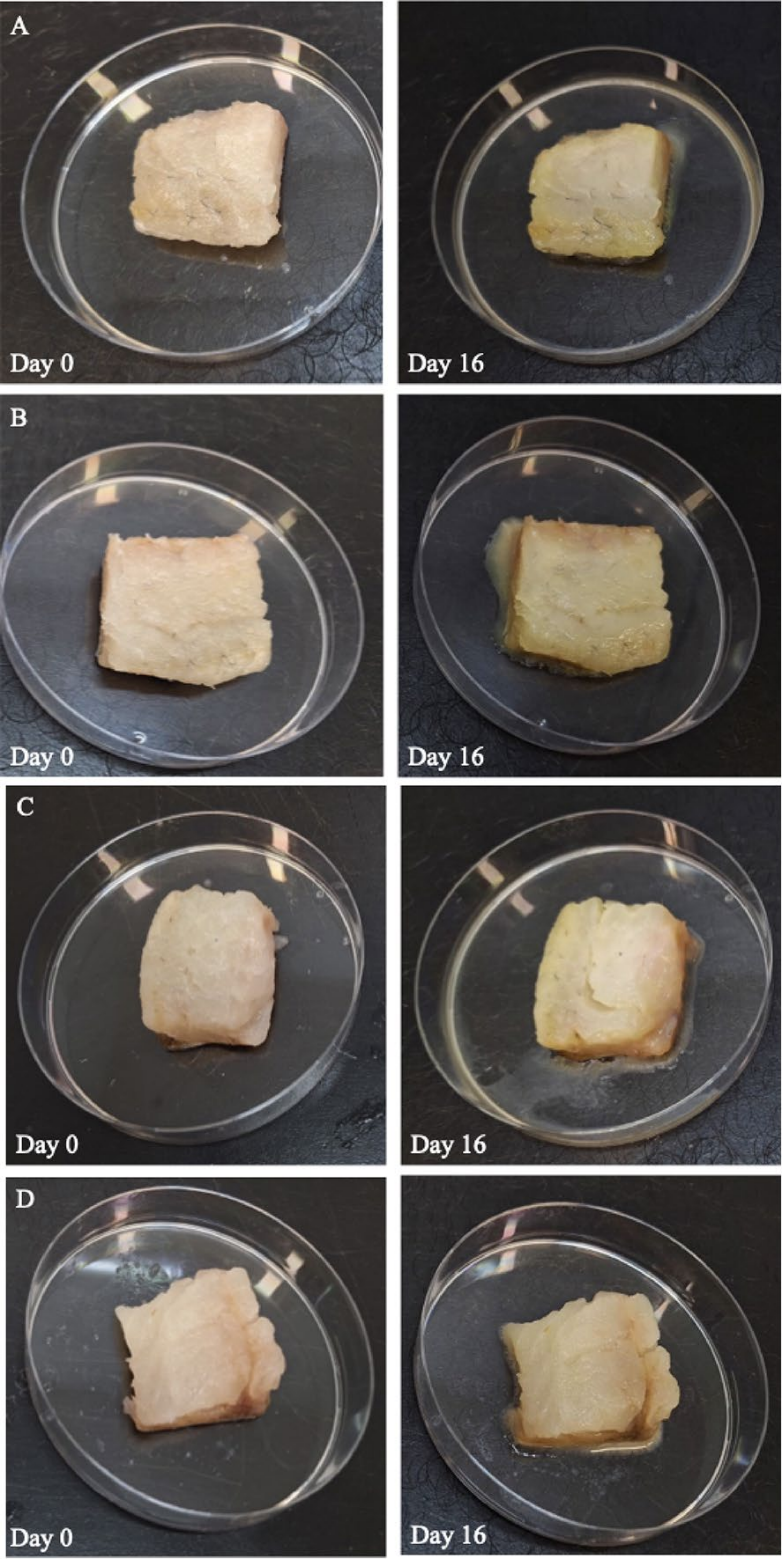
Visual appearance of packed gilthead seabream fillets with the developed packaging films (**Α**) PVC, (**B**) Pectin-pectin (**C**) HPMC-gelatin and (**D**) HPMC-pectin.

**Figure 5:**
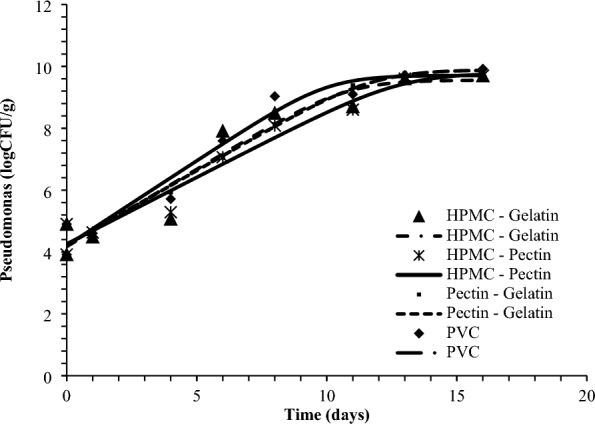
*Pseudomonas* spp. growth in seabream fillets packed with composite films based on pectin, gelatin, and HPMC when stored at 2 °C compared with seabream fillets packed with PVC films.

**Figure 6:**
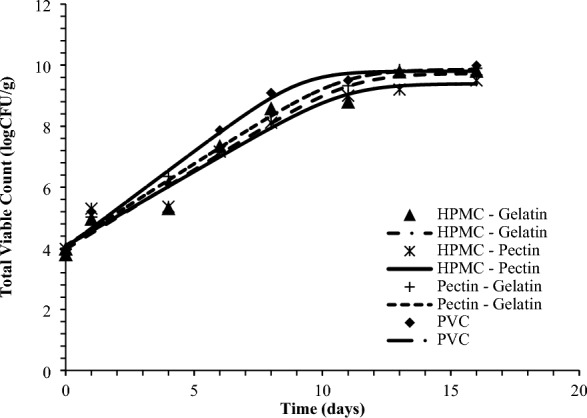
Total Viable count in seabream fillets packed with composite films based on pectin, gelatin, and HPMC when stored at 2 °C compared with seabream fillets packed with PVC films.

Figure 4 represents the visual appearance of gilthead seabream fillets packed with the developed packaging films, at the day 0 and after 16 days of storage at 2 °C. The initial load of *Pseudomonas* spp. and Total Viable Count (TVC) was 4.10 ± 0.32 and 3.97 ± 0.33 logCFU/g, respectively. Figures 5 and 6 represent the growth curves of *Pseudomonas* spp. and TVC fitted to Baranyi Growth Model. For all tested films, no lag phase was observed. No significant differences were observed in microbial growth rates between the different tested packaging films (Table 7). The final microbial populations were 9.6–9.7 for *Pseudomonas* spp. and 9.7–10.7 for TVC. Considering the limit of acceptability of 10^7^ CFU/g for TVC, the shelf-life of gilthead seabream fillets at 2°C was calculated^49^. Based on this limit, the shelf-life of gilthead seabream fillets was determined as 7–8 days at 2 °C in all tested packaging conditions, which is in agreement with the commercial use-by-date set for this product, while also with other studies in the literature evaluation the shelf-life of Mediterranean fish fillets at 0–2 °C^49–51^.

**Table 7:** Kinetic parameters of microbial growth (growth rate, k; maximum population; N_max_) for *Pseudomonas* spp. and TVC in gilthead seabream fillets packed with composite films based on pectin, gelatin, and HPMC.

	k (day^−1^)	N_max_ (logCFU/g)	R^2^
*Pseudomonas* spp.			
PVC	0.5494 ± 0.0706^b^	9.706 ± 0.343^a^	0.9545
Pectin-HPMC	0.4308 ± 0.0484^a^	9.789 ± 0.496^a^	0.9558
Pectin-gelatin	0.4817 ± 0.0393^b^	9.888 ± 0.296^a^	0.9789
HPMC-gelatin	0.4996 ± 0.0889^b^	9.555 ± 0.515^a^	0.9131
Total viable count			
PVC	0.6295 ± 0.0496^b^	9.801 ± 0.205^b^	0.9826
Pectin-HPMC	0.4880 ± 0.0559^a^	9.390 ± 0.335^a^	0.9622
Pectin-gelatin	0.5359 ± 0.0301^a^	9.859 ± 0.174^b^	0.9908
HPMC-gelatin	0.5197 ± 0.0599^a^	9.728 ± 0.376^b^	0.9612

^a,b^Different superscripts in the same column indicate statistically significant differences.

The results showed that the edible packaging materials developed by pectin-gelatin, HPMC-pectin, and HPMC-gelatin may adequately reduce the use of petroleum-based polymers, such as polyvinyl chloride, and maintain a high quality of gilthead seabream fillets without affecting fish microbial spoilage and thus the shelf-life. According to the literature, gelatin-based films have been used in order to preserve animal-based products. Roy et al. produced gelatin/ager based films enhanced with antibacterial and antioxidant compounds used to pack meat over 8 days at 10 °C. The results showed that pork belly meat packed with alternative material had slower growth of total aerobic bacterial count^52^. Alirezalu et al. synthesized active chitosan films (1%) and the films used to test the effects of packed beef fillets at 4 °C for 12 days. Packed meat with chitosan films had lower TVC indicating better preservation of packed meat compared to LDPE films (7.38 ± 0.16 and 8.83 ± 0.09 logCFU/g at 12 days, respectively)^53^. Another research (Gulzar et al.) enhanced PLA films with gelatin/chitosan solutions incorporated with various concentrations of nisin. The produced PLA films coated with gelatin/chitosan and 0.4% nisin used to pack Asian seabass sliced to determine the microbial and chemical changes of stored fish at 4 °C for 12 days. The initial load of TVC of the fish packed with LDPE films and PLA-gelatin/chitosan/nisin ranged from 4.23 to 4.30 logCFU/g. In 3 days, the TVC for packed fish with LDPE was higher (6.46 logCFU/g) compared to packed fish with PLA-gelatin/chitosan/nisin (5.31 logCFU/g), indicating that the composite films preserved fish more adequately than single LDPE during refrigerated storage^54^.

The aim of the study was to replace the use of petroleum based materials without reducing the shelf-life of the packed fish. Pectin, HPMC and gelatin films do not exhibit antimicrobial property activity, if no antimicrobial compounds have been incorporated in the matrix of biopolymers, but they can help to release a wide range of active compounds^55^. Other studies investigated the antimicrobial activity of the films when combined with antimicrobial compounds. Shivangi et al. studied the antimicrobial activity of pectin films against *Pseudomonas aeruginosa* and *Bacillus cereus,* and they pointed antimicrobial activity against the two microorganisms only when crude mulberry leaf extract, deoxynojirimycin and chlorogenic acid were added. In the case of pure pectin films without extracts no activity against the microorganisms was observed^24^. According to Möller et al., HPMC exhibits antimicrobial activity when it is combined with chitosan^56^. Leite et al., tested the antimicrobial activity of gelatin films and they pointed that the films without nonoxidized tannic acid (nTA) had not inhibitory effect against *Staphylococcus aureus* and *Escherichia coli* growth^57^.

Future studies should be conducted focusing on the incorporation of antimicrobial compounds such as essential oils, Zn ions, or silver nanoparticles (AgNPs), with the aim to address the effects of using active packaging materials on the shelf-life of gilthead seabream fillets.

## Conclusions

Alternative edible and biodegradable food packaging films were developed using pectin, gelatin, and HPMC which could replace petroleum-based packaging materials, such as PVC. All the tested films showed excellent barrier properties in the UVC wavelengths and good performance in the UVA and UVB wavelengths range. The obtained results showed the potential of synthesized films made from polysaccharides and proteins (HPMC, pectin and gelatin) as green environmentally friendly packaging materials to adequately preserve fresh gilthead seabream fillets during refrigerated storage at 2°C, in terms of microbial spoilage.

## Methods

### Materials

Hydroxypropyl methylcellulose (hydroxypropyl 5–8%, methoxy 28–30%) (2.6–5.6 Pa-s, 2 % in H_2_O 20 °C) was supplied from ACEF SPA (Piacenza, Italy). Low methoxy amidated pectin with a degree of esterification 30–36% and degree of amidation 14–20% was purchased from Herbstreith & Fox (Neuenbürg/Württ, Germany). Gelatin was provided by AppliChem GmbH (Darmstadt, Germany) with viscosity higher than 6000 cP. Glycerol (≥ 99.5 %) was obtained from Sigma-Aldrich (St. Louis, USA). The gilthead seabream (*Sparus aurata*) fillets used for the shelf-life test were obtained from AVRAMAR SA (Greece).

### Preparation and characterization of films

The films were produced according to the solvent casting method. 2% w/v of each biopolymer was dissolved into water and mixed for 30 min. Gelatin and pectin solution was stirred under 35 °C and the HPMC solution was stirred under 80 °C for 30 min. Then, glycerol was added at 30% of the weight of each biopolymer and mixed for 15 min. The HPMC solution was stirred for 1 h and the temperature was decreased to 35 °C before adding glycerol to increase the transparency of the films. The solutions were mixed in proportions of 1:1 and three additional types of film-forming solution were produced. In total, six different types of film-forming solutions were produced, three based on one biopolymer (pectin, HPMC, gelatin) and three composites (pectin-HPMC, pectin-gelatin, HPMC-gelatin). All solutions were centrifugated at 4000 rpm, 25 °C for 10 min. After centrifugation 20 g of each solution was poured into plastic petri dishes with diameter 14 mm^19^.

#### Rheological properties

The measurements of the steady shear rheological properties of the film-forming dispersions (flow curves) were performed in an extended shear rate range (1–1000 s^−1^) at 25 °C. Α stress-controlled rheometer (Discovery HR-3, TA Instruments, New Castle, DE, USA) was used, equipped with a plate-plate geometry (40 mm diameter) with a gap set at 1000 mm. Equilibration was performed by leaving the samples on the geometry for 2 min. Next, steady shear properties were evaluated. All measurements were made in triplicate and the presented rheological values are the average of results. The viscosity of each primary solution was 28.55 ± 1.92 mPa s for gelatin, 36.06 ± 0.09 mPa s for pectin solution and 3398.55 ± 97.43 mPa s for HPMC solution.

#### FT-IR spectroscopy

All spectra were recorder using an IROS-05 FTIR spectrophotometer (Ostec corporation group, Russia) equipped with diamond crystal and a Mercury-Cadmium-Telluride (MCT). The recording spectral range was from 4000 to 400 cm^−1^ at a resolution of 4 cm^−1^ and 64 scans. The background spectrum was obtained using the pure diamond crystal. The spectrum of each sample was recorded in triplicate using a different sub-sample each time. Then, each spectrum was manipulated using the corresponding functions of the software (OMNIC ver. 8.2.0.387; Thermo Fisher Scientific Inc., Waltham, MA, USA) as follows: each spectrum was “automatically smoothed”, using the Savitzky–Golay algorithm (2^nd^ order, 5-point window), baseline corrected using the “automatic baseline correction” (2^nd^ order polynomial fit) and the averaged spectrum of each example triplet spectra was calculated.

#### Film thickness

Film thickness was measured with a digital micrometer (IP65, SAMA Tools, Viareggio, Lucca, Italy) at five different randomly chosen positions. The average and standard deviation of five measurements were recorded.

#### Mechanical properties

The mechanical properties were determined using a dynamometer (Zwick/Roell, Ulm, Germany). The results were analyzed by using the software TestXpert®II (V3.31). Elastic modulus, tensile strength and elongation at break were the three mechanical properties that were analyzed. ASTM D882 (ASTM, 2001) method was followed^58^. Films were cut into rectangular strips (9 × 1.5 cm^2^). Crosshead speed was 50 mm/min, and the initial grip separation was 70 mm.

#### Water vapor transmission rate and water vapor permeability

The WVTR and WVP were determined gravimetrically, according to ASTM E96 method with slight modification. Films were sealed on top of glass vials. Vials containing 2 g anhydrous CaCl2 in order to achieve 0% RH and were placed into a desiccator with BaCl2 (90% RH). The desiccator was isothermally stored at 40°C for 10 days. WVTR was calculated according to the Eq. (1):

1 $${\text{WVTR }} = { }\frac{\Delta W}{{\Delta t}} \times A$$

where $\frac{\Delta W}{\Delta t}$ is the measuring weight of the vials per day (g/day) and A is the surface of the film (m^2^). The units of the WVTR are $\frac{g}{day}$× m^2^. WVP is calculated according to the Eq. (2):

2 $${\text{WVP }} = {\text{ WVTR}} \times \frac{L}{\Delta P}$$

where L is the average of the film thickness (mm) and ΔP is the difference of the vapor pressure between the two sides of the film (kPa). An average of 5 measurements of each film were used^59^.

#### Moisture content

The moisture content (MC) of the films was determined by gravimetric method after drying them at 105 °C for 24 h. The initial weight of the film was measured (Wi), and the dry film was measured (Wf), as well. Five replicates were analyzed. The moisture content was calculated according to the Eq. (3): the results was expressed as g of water per 100 g of dry film.

3 $${\text{MC }} = { }\frac{Wi - Wf}{{Wi}} \times 100$$

#### Water solubility

The films were cut of 2 × 3 cm^2^ pieces and were kept in a desiccator with dried silica to achieve 0% RH, for 7 days. The films were weighted in the analytical scale and placed into falcons with 50 mL distilled water. The falcons were agitated with vortex for 1 h at room temperature. The remained pieces of the films were dried in the air oven at 50 °C until constant weight. The water solubility (WS) is calculated according to the Eq. (4):

4 $${\text{WS }} = { }\frac{Wi - Wf}{{Wi}} \times 100$$

where Wi is the initial dry weight of the samples and Wf is the final dry weight of the samples. Four replicates were conducted for each type of film.

#### Contact angle

Static contact angles of the films were measured by using Theta Flow Optical Tensiometer) Biolin Scientific, Gothenburg, Sweden) according to ASTM D5946 method. The sessile drop technique was used at room temperature in air. Four microliters of distilled water were dropped on the surface of the materials and the contact angle was measured as a mean of 12 different points.

#### Color properties

The color parameters (L, a, b) were defined by CR-400 Minolta colorimeter (Minolta Camera, Co., Ltd., Osaka, Japan) with D65 illuminant and 10° observer angle. Calibration of the instrument was executed with white standard (L* = 99.36, a = − 0.12 and b = − 0.06). The total color variation (ΔE) of all films was calculated according to the Eq. (5):

5 $$\Delta {\text{{\rm E} }} = { }\sqrt {L^{2} + a^{2} + b^{2} } ,$$

where ΔL, Δa, and Δb are the differences between the value of the film and the white standard. An average of 12 measurements of each film were used.

#### UV–Vis barrier properties

The UV–Vis barrier properties were measured by spectrophotometer (VWR ® Douple Beam UV × Vis 6300 PC spectrophotometer, China) at 190–800 nm wavelength range. Film samples (3 × 3 cm^2^) were put on a paper frame used as a support. An average of 3 measurements of each film were calculated. The absorbance and transmittance values were recorded. The absorbance and transmittance were evaluated at specific wavelengths, 200, 280, 350, 400, 500, 600, 700 and 800 nm.

#### Microbiological evaluation of packed fish

The three main types of the developed films: HPMC-gelatin, HPMC-pectin, and pectin-gelatin, were tested for their applicability as packaging materials of fresh gilthead sea bream (*Sparus aurata*) fillets. Fish fillets were cut in rectangular slices (12 ± 2 g each) and placed into conventional rigid polyethylene terephthalate (PET) containers, which were sealed using the developed biobased films, in sterile conditions. The edible films were applied as alternatives to the conventional flexible covers of the PET trays carrying fresh gilthead seabream fillets in the market, so the experiment was carried out at the actual storage conditions of fresh fish fillets. Six replicates were produced for each type of film. For microbiological analysis, 10 g of fish was collected from each container and placed into a sterile stomacher bag with 90 mL sterilized Ringer solution (Merck, Darmstadt, Germany) and homogenized for 60 s with a Stomacher (BagMixer®, Interscience, Saint-Nom-la-Breteche, France). Zero point one milliliters of tenfold serial dilutions of fish homogenates were spread on the surface of appropriate media in Petri dishes for microbial enumeration. Total aerobic viable count (TVC) was enumerated on Plate Count Agar (PCA; Neogen, Lansing, MI, USA) after incubation at 25 °C for 72 h. Pseudomonas spp. Were enumerated on Cetrimide Agar (CFC; Condalab, Torrejon De Ardoz, Spain) after incubation at 25 °C for 48 h. For each sample, three dilutions per sampling time were enumerated^49^. The microbial growth was modelled using Baranyi growth model (Baranyi and Roberts, 1995). For curve fitting the program DMFit (IFR, Institute of Food Research, Reading, UK) was used (available at http://www.combase.cc/index.php/en/).

#### Statical analysis

Statical analysis was conducted by one-way analysis of variance (ANOVA) followed by Tukey’s multiple range test (p < 0.05) with SPSS statical program (SPSS 20 for Windows, SPSS INC., IBM, New York). The results were expressed as mean ± standard deviation (SD).

## Data Availability

The datasets used and/or analysed during the current study are available from the corresponding author on reasonable request. The datasets are part of ongoing scientific project and the respective deliverables have not been yet completed.
